# Integrating Digital Innovation Mechanisms in Digital Infrastructures: The Case of Digital Remote Care

**DOI:** 10.1177/11786329231200704

**Published:** 2023-09-26

**Authors:** Anne KS Ajer, Egil Øvrelid

**Affiliations:** Department of Informatics, University of Oslo, Oslo, Norway

**Keywords:** Digital innovation, digital infrastructure, digital remote care, e-Health, hospital

## Abstract

Digital innovation (DIN) is crucial for managing the growth of resource use in the hospital sector and for providing citizens with services aligned with the requirements of the modern world. DIN includes the co-creation of novel services, such as digital remote care (DRC) solutions. The healthcare sector, with a plethora of applications, is an example of a large digital infrastructure. Our study aims to explore how DRC initiatives can be integrated in large-scale digital infrastructures. Our in-depth case study, which explores 72 different DRC trajectories at 9 hospital health trusts in Norway, reveals the dynamic interplay among 3 key mechanisms – idealistic entrepreneurship, anchoring and remote infrastructure. Our contribution to the DIN literature is a model that shows the interplay among these key mechanisms, which increases the innovation pace, improves the innovations’ scalability and provides a robust organisation that constantly implements innovations. As a contributions to DRC practice, lessons learned to speed up the innovation pace are offered: (1) Create a DRC organisational structure. (2) Ensure financial predictability. (3) secure anchoring upward in the governance structure. (4) Make the remote infrastructure appropriate for integration with the current digital infrastructure. (5) Advocate the success across the organisation to spur others to innovate.

## Background

Since the beginning of the 21st century, the growth in the number of patients due to an ageing population, together with the rise in multi-morbid patients with complex needs and high expectations from the citizens related to health care have imposed new burdens on the hospital. Addressing this emerging situation is difficult for the hospital in its existing institutional form.^1,2^ Even if technology has made it possible for many treatments (that previously required hospitalisation) to be performed on outpatients,^3^ there is a politically driven desire for more treatments to be administered in patients’ homes.^4^ Thus, there is a need to relieve the workload of the geographic location-based hospital institution and find new approaches to deliver care services. Such strategies should aim to reduce the demand for both financial and human resources,^4,5^ as well as take advantage of technology to alleviate physicians’ burden of handling less demanding patients or tasks, so they can focus on proactive care.^6^ Many researchers have paid attention to addressing these issues, but they remain valid research areas.^5,7,8^

Fortunately, digital technology provides new opportunities, such as digital remote care (DRC).^9^ In this study’s context, DRC refers to the activities performed when follow-up with a patient is moved outside a healthcare institution and enabled through digital systems. Different terms are used for this concept that refers to patient care outside a healthcare institution, enabled by digital technologies, for example *digital and remote care*,^10^ *digitally enabled remote care, e-health, telehealth* and *telemedicine.*^11,12^ Since the dialogue between the patient and the caregiver can be held in places other than the patient’s home, we suggest using the term *digital remote care (DRC)*. According to the definition provided by the Directorate of Health (p. 8),^13^ DRC ‘includes the activities/actions that enable [. . .] the patient[s], outside the traditional arenas where patients meet healthcare professionals, [to] acquire, register and share clinically relevant information about their state of health electronically, for the purpose of providing information or guidance for [their] self-mastery, and/or [to] provide decision support [for] diagnosis, treatment or follow-up [by] healthcare professionals’. This definition covers the meaning that we have found in our empirical data. Some patients are supposed to be inpatients but stay home, but most DRC solutions are for those who normally go to hospitals for regular follow-ups. Many DRC solutions seek to support the whole care pathway. ‘Care pathways are generic instruments, designed to manage a patient with a particular disease or condition (e.g. stroke)’ (p. 182).^14^

However, moving services out of the hospital is a challenging endeavour.^9^ This require the introduction of a new governance regimes with new organisational structures to prepare the new offerings, facilitate faster decision making, empower the employees to innovate and empower the vendors to participate, etc. Information security, safe treatment and treatment quality outside the hospital are also areas to address.^15,16^ Moreover, ensuring smooth information flows (interoperability) across the information and communication technology (ICT) landscape is not only pivotal for healthcare delivery but also quite challenging.^17^ Interoperability can be achieved through standards, but this has proven to be difficult due to different interoperability standards for the same type of health information systems,^18^ additionally ‘medical professionals are confined to their specialisations, following clinical protocols to address specific clinical needs’ (p. 16).^19^

Throughout the 20th century, increasingly sophisticated technologies has been developed to support and strengthen various treatment trajectories. We frame these large portfolios of clinical IT systems as digital infrastructures,^20^ which offer a huge potential to understand this complex sociotechnical healthcare environment.^21,22^ The central object of digital infrastructure research comprises the interconnected networks of organisations, people and technologies. Hanseth and Lyytinen (p. 1)^23^ define it as ‘a shared, open (and unbounded), heterogeneous and evolving socio-technical system (which we call installed base) consisting of a set of IT capabilities and their users, operations, and design’. The heterogeneous mix of people and technologies is built on the installed base. According to the infrastructure theory, the installed base can be expanded through bottom-up strategies.^24^ Another way of framing innovation in digital infrastructure is through lightweight IT.^25^ Lightweight IT focuses on how innovation in such infrastructures can occur by adding new modules or replacing existing functionality with new apps.^26^ Innovation in large-scale infrastructures is demanding, but Bygstad and Øvrelid^27^ have shown how lightweight IT can enable faster implementation and deployment of digital innovation (DIN).

In our study, we use the definition of DIN ‘as the co-creation of novel offerings through the recombination of digital and/or physical components’ (p. 361).^28^ We conjecture novel offerings as new digital systems and new work processes, and we include human beings in physical components. The digital aspect has properties that allow recombination and value co-creation that go beyond traditional innovation; in this regard, more research is needed.^28^ The information system (IS) community has paid attention to the role of digital technology itself,^29^ and the tactics that entrepreneurs can employ to pursue DIN have been described.^30^ However, researchers should examine how DIN influences the organisations so they can manage the complexity of what DIN entails, for example, cutting across architectural layers (such as contents, service, network and device), building new collaboration and new governance structures to align with emerging supra-organisational forms, such as platforms.^28^ Noteworthy is that research indicates that neither centralised nor decentralised governance regimes are ideal because strong centralisation leads to innovation fatigue^31^ and strong decentralisation likely results in systems with no scaling capacity.^32^

Digital innovation is closely related to entrepreneurship, thus we bring in institutional entrepreneurs as essential actors with sufficient power and interest to create new organisational structures and work practices.^33^ The actors can be organisations, groups of organisations, individuals, or groups of individuals.^34^ In our context, it is imperative for the institutional entrepreneur to have the ‘imaginative projection’ capability to understand how digital technology can generate new solutions (p. 942).^30^ Moreover, there is a need for more research to understand the prerequisites for an ordinary employee’s participation in DIN.^35^

Examining the emergent local DRC solutions reveals them as user-oriented lightweight IT systems that support a faster innovation pace than that of heavyweight information systems, which are typically the stable core business systems that have a slow innovation pace.^27,36^ Nevertheless, the solutions usually require interaction with the established digital infrastructure, including the people who use and govern the solutions.

In this paper, we frame the process of introducing and integrating this new DRC regime into the digital infrastructure as a challenge.^37^ Prior relevant studies have mostly reported results from studies limited to one patient group, for example, Braune et al,^10^ Kaye et al^11^ and Bagot et al^38^ or mainly video technology, for example, Wade et al^8^ Alami et al.^39^ Thus, we explore the emergence of diverse DRC solutions in 72 different initiatives under the auspices of 9 local hospital health trusts (HTs) in a large hospital region in Norway. Our research question is as follows: *How can a DRC initiative be integrated in a large-scale digital infrastructure?*

## Method

### Study design

This research is designed as a qualitative case study of the phenomena of DIN practices related to DRC in the hospital sector. By investigating 9 somatic hospital HTs we identified 72 different DRC initiatives and explored these through document reviews and interviews. This enabled us to compare the different HTs, and we obtain rich data to understand how DIN practices have emerged.^40^ This study is a part of the project ‘architecture governance in the health sector’. The project is reviewed and approved by the Norwegian Center for Research Data (reference number: 372461).

### Study site

In Norway, hospitals are public and organised as HTs allocated to a regional health authority. We study the South Eastern Region Health Authority (SERHA), with 11 HTs (9 somatic, 1 for purchasing and 1 for ICT operations) that had a total of 81 000 employees and an annual turnover of 88.5 billion NOK in 2020. The SERHA’s centralised technological department’s scope of responsibilities includes strategy, the overall architecture and compliance issues, among others. In the region, a large digital infrastructure has evolved over time, including 1250 unique applications as of 2022, ranging from large organisational ISs to smaller specialised apps for certain units. Investments in ICT are made at the regional level and to some degree at the local levels. The need for care services at home^4,41^ has obliged the hospitals to explore how DIN can change the ways of delivering patient services.

DRC is not a new topic for the region, but has limited scope and distribution.^39^ Nevertheless, the COVID-19 pandemic has been a catalyst for clinicians’ and healthcare managers’ awakening about the worthiness of DRC. Traditionally, the SERHA has centralised IS governance, with limited autonomy for the HTs. However, the SERHA currently lacks central arrangements to govern DRC initiatives, but according to anecdotal evidence, local initiatives suffer from their limited capability to reach the production stage and the necessary capability to scale. Thus, we have found it timely to examine the conditions required for a local DRC initiative to progress from idea to production and how they are governed.

### Research approach and quality assurance

Our philosophical paradigm belongs to critical realism, where ‘a causal explanation for a given phenomenon is inferred by explicitly identifying the means by which structural entities and contextual conditions interact to generate a given set of events’ (p. 787).^42^ To gain insights into a phenomenon, critical researchers can apply principles from interpretive research, as outlined by Klein and Myers,^43^ but they are insufficient.^44^ Therefore, we also followed the principles proposed by Wynn and Williams^42^ when we identified the key mechanisms and their relationships and assessed their explanatory power. To support our theoretical understanding, we used Pawson and Tilley’s^45^ context–mechanism–outcome approach as a framework. Importantly, the research process, with data collection, analysis, and theory development, was iterative and proceeded in parallel.^42^

Since the analysis was conducted during the data collection period, we used the opportunity to discuss the results with the interviewees and thus enhance our understanding, in accordance with the principle of interaction between the researchers and the subjects.^43^ As we had conducted research related to the architecture and evolution of new systems in the SERHA for many years, we could apply Klein and Myers’^43^ principle of contextualisation. Furthermore, we discussed the data and searched for alternative explanations and interpretations, stemming from the interpretations and potential bias of the sources. In this way, we followed Klein and Myers’^43^ quality principles of multiple interpretations and suspicion, while adhering to Wynn and Williams’^42^ principles of explication of structure and context, as well as retroduction, to develop the theoretical model. Table 1 provides an overview of the research process and its outcome.

### Study outcome

**Table 1.** The research process and its outcome.

Activity	Outcome
Reading secondary data, eg, strategy reports, investigations and web pages related to digital remote care (DRC)	DRC is a national strategic goal
	DRC is not centrally governed but seems coincidently scattered throughout the region
	Insight into some of the DRC initiatives
Conducting interviews and meetings, primarily with the technical personnel	Confirms our suspicions from the secondary data Reveals different strategies and governance structures for DRC
Attending a conference, with DRC as a theme	Improved insight into the various DRC initiatives
Coding and structuring the findings	Patterns for different strategies emerge, and innovation seems connected to specific persons
Conducting interviews and meetings, primarily with clinicians	Reveals the exceptional efforts of the DRC initiators, their challenges related to management support and financial issues and to integration with existing systems
Discussing emerging patterns of the differences among the HTs, related to speed of innovation	Idealistic entrepreneurship, anchoring and remote infrastructure were identified as key mechanisms
Developing a theoretical model using the context–mechanism–outcome approach^45^	Contextual conditions lead to DRC initiatives, and when the key mechanisms are in place, the organisation is aligned with the national goal to foster further digital innovation in the healthcare sector

### Data collection

First, we collected secondary data. Since the HTs have obligations to implement the national and the regional strategies, we collected secondary data at both national and regional levels. These comprised strategy documents and reports, including internal documents on regional DRC strategy, which totalled 49 documents. Furthermore, we searched the hospitals’ websites and a few other relevant websites, using DRC and similar keywords to identify initiatives across the region. In sum, we found 47 web pages explaining the DRC initiatives in detail; the aim, the status and the expected benefits.

The primary data for this study come from interviews with informants from the 9 somatic HTs in the SERHA, from the central SERHA, and from speakers at a conference. In this paper, the HTs are anonymised (using the names of gems). We decided to use a purposeful sampling strategy to ‘identify people with great knowledge and/or influence (by reputation) who can shed light on the inquiry issues’ (p. 268).^40^ First, we contacted the chief information officer (CIO) at each of the 9 somatic HTs; most of them agreed to be interviewed, but some forwarded our request to a relevant employee. Furthermore, we contacted the HT representatives identified in the secondary data and at the conference. The interviewees’ positions included CIOs, different kinds of managers (eg, innovation, section, team), special advisors and project or programme managers. Most of the interviewees were educated either as nurses or physicians, while the CIOs had IT and/or management as their educational background.

We conducted 16 semi-structured and open-ended interviews and 2 meetings with 20 individuals from October 2021 to March 2022, which were held as video conferences. On average, the interviews lasted 45 minutes, and the meetings took 100 minutes. We inquired about strategies for DRC at the HT and how DRC was organised and governed. Furthermore, we asked about details for each DRC initiative – which patient groups were involved, how the initiative was started, its status, who was involved in its development, the technical side (eg, vendors and integration), and the organisation’s view on the initiative, anchoring, financing and benefits. Finally, we asked about the major challenges to the initiative and the major success factors for the initiative to get started. We soon discovered major differences across the HTs in terms of the number of DRC initiatives and how they approached the phenomenon; thus, we adapted our interview protocol accordingly.

After obtaining the participants’ consent, we recorded and transcribed all interviews, except one, where we took notes instead.

In November 2021, a national conference was held, where many sessions had DRC as a theme. The sessions were available online afterwards, and 16 relevant speeches (ranging from 5 to 20 minutes) were transcribed, while notes from some speeches and debates were made. We also used the opportunity to ask the speakers some questions and agreed to hold a follow-up meeting at a later date.

### Data analysis

Both the secondary and the primary data were coded in NVivo (a tool for qualitative data analysis), and we used spreadsheets for overviews and for pattern recognition. Furthermore, we used Pawson and Tilley’s^45^ context–mechanism–outcome approach to develop our theoretical understanding because it ‘allows for the analysis of possible configurations of mechanisms and relevant context-variation to explain a particular outcome’ (p. 6).^25^

We continued to compare the data and to discuss the HTs’ common characteristics related to the initiatives. We observed that some HTs were more successful than others in terms of more productive DRC solutions, and we wanted to identify the main reasons for this. Through a retroductive process, we identified around 10 mechanisms that could partly explain the success, and after reflecting on the explanatory power related to the empirical evidence of each of them, we were left with 3 key mechanisms.^46^ Part of the analysis consisted of comparing the number of initiatives at each HT and finding out if they had purchased a platform that was ready to be integrated with the core systems and whether they had an organisation to support and advocate DRC initiatives (see Table 2).

**Table 2.** Overview of the 9 somatic health trusts (HTs) and their status related to digital remote care (DRC) initiatives.

HT	No. of initiatives and status				Purchased platform ready for integration	DRC – Organisation
	Pr	Pi	T	Pl		
HT1 (Emerald)	12	2		2	Y	Y
HT2 (Opal)	6	2	1	3	Y	Y
HT3 (Jade)	5	5		3	N	Y
HT4	6	2	2		N	Partly
HT5	3	1		2	Y	Partly
HT6	2	2	1		N	N
HT7	3	1	1		N	N
HT8	1	2			N	N
HT9 (Topaz)	1		1		N	N
Total	39	17	6	10		

Abbreviations: Pi, pilot; Pl, planned; Pr, production; T, terminated.

## Results

**Figure 1.**
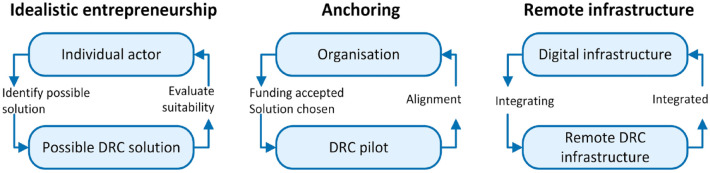
Illustration of the connection between the social and the technical aspects of each mechanism.

We have observed similarities across the 9 hospital HTs in how the dynamics among the 3 key mechanisms spur innovation. These mechanisms were recurrent and were assessed to be of prominent significance regarding an initiative’s ultimate fate. Table 3 and Figure 1 respectively describe and illustrate the 3 key mechanisms and how they are connected with the sociotechnical digital infrastructure. First, Table 2 presents an overview of the findings related to the number of DRC initiatives across the HTs. Next, Table 3 presents the 3 key mechanisms with corresponding Figure 1, followed by a description of the mechanisms in detail. Furthermore, 3 empirical cases illustrate the importance of and the interrelations among the key mechanisms for a DRC initiative to be integrated into a large-scale digital infrastructure. The cases had varying outcomes, depending on the dynamics among the mechanisms (see Table 4).

**Table 3.** Definitions of key mechanisms for digital innovation in a large user organisation.

Mechanism	Definition
Idealistic entrepreneurship	A process where an individual unit identifies and evaluates a possible DRC solution
Anchoring	A process where funding is accepted and a DRC solution is chosen to align with the organisation
Remote infrastructure	A process of integrating a remote DRC solution into a digital infrastructure
	This mechanism requires anchoring

**Table 4.** Matrix showing the presence or absence of the 3 key mechanisms for digital innovation.

Case	Idealistic entrepreneurship	Anchoring	Remote infrastructure (RI)	Outcome
1: Topaz	*Present* A clinician advances a project to the pilot phase with clients onboard	*Absent* No internal budget or organisational structure for the digital remote care (DRC) initiative	*Absent* The RI needed integration, which was not accomplished	*No innovation* Project cancelled, DRC innovation put on hold
2: Jade	*Present* Several dispersed initiatives Some clinicians stand out as driving forces	*Present* A specific DRC organisation, organised as a project	*Absent* Different choices of needed systems and integration were only partially accomplished	*Slow innovation pace* Struggled to have initiatives started or to advance from pilot phase to production
3: Emerald	*Present* Both clinicians and administrative personnel stand out as idealistic entrepreneurs	*Present* A specific DRC organisation, organised as a programme	*Present* A choice of one RI from which to innovate, which has sufficient integration	*Steady innovation* Continuous new initiatives in production

### Digital innovation mechanisms

The first mechanism is *idealistic entrepreneurship*. All of the DRC initiatives we found were typically based on an idea from a practising clinician, who was usually the one who managed the project and made substantial efforts beyond ordinary work hours to achieve its progress. It also implied types of work and knowledge outside one’s profession.

The second mechanism, *anchoring*, involves gaining top management support and organisational alignment. Anchoring can take the form of local funding, approval of resource requisition or approval of new organisational structures to support a DRC initiative. Some support structures are organised into a programme, and others involve a team in a line organisation. Anchoring also implies the organisation’s capabilities to grasp the momentum of using the advantages that lightweight IT brings to the organisation, understand the challenges faced by a single actor and facilitate support to make innovation happen. Ultimately, there is an alignment of the organisation with the national goal to focus on innovation through DRC solutions.

The third mechanism is *remote infrastructure*, so the new solutions can adapt to the organisation’s current digital infrastructure. This includes sufficient integration and must be approved by the organisational unit that governs the integration.

### Case 1: Topaz HT

Topaz HT is one of the smallest HTs in the region. Besides the use of video consultations, they had one local initiative, which was terminated. The empirical data show how an idealistic entrepreneur is a driving force, but when the other mechanisms are absent, the initiative has little chance of survival. It is worth mentioning that at the end of our data collection, Topaz implemented a centrally governed DRC solution for psychiatry.

#### Idealistic entrepreneurship

The initiative was related to digital support for home dialysis and was started by a physician and embraced by the nurses and the patients.

It was one of the kidney doctors who was very interested to explore new technology [. . .], then we had a dialogue with [a local company engaged in healthcare and ICT] if we could come up with something together around home dialysis patients (development director).

According to the development director, it is imperative that the professional communities are interested and want the change since they are the ones that drive the development. In this case, represented by a physician as an idealistic entrepreneur who started the process.

#### Anchoring and remote infrastructure

Notwithstanding goodwill and help from the development department, the initiative was terminated. The development director explains the main reasons:

They [clinicians] still had to do some double work, double registrations, because we did not have integration, either to the patient journal or to the system for ordering dialysis fluid. [. . .] We got very limited funding for the project. We have a director who was the former finance director, and I am responsible for innovation, but I have zero NOK in the budget. Funding has to be applied for separately for each innovation initiative (development director).

To achieve proper integration and connect the remote infrastructure to the existing one, the Hospital Partner (the operational ICT unit in SERHA) must be involved. The director told us that they had a dialogue, but the service would be too expensive, given the zero budget, and the Hospital Partner was not eager to have yet another system to support.

The Topaz case shows that idealistic entrepreneurship is in place, but lack of anchoring and lack of remote infrastructure terminated the initiative.

### Case 2: Jade HT

Jade HT is one of the largest HTs in the region. It has many DRC initiatives, but most of them are still projects in the pilot phase or not yet started at all. Many have external funding but have several obstacles to overcome before eventually going into production.

#### Idealistic entrepreneurship

One system, related to an asthma tuner, is in production in one clinic. The initiative is managed by a physician. When the pilot phase was successful, the physician asked the Jade clinic manager to use the system in the hospital, which the latter approved.

The asthma tuner is a small project, but there are complicated processes before it can go live, and it can be exhausting for those who operate the project. The following statement illustrates some of the unforeseen tasks that can confront an idealistic entrepreneur:

I started the method evaluation in February–March 2021 and finished it in May [. . .]. It was a big job with a structured literature review, economic evaluation, and risk and vulnerability analysis. I had not anticipated it [all the work]; it was much more than I had imagined, so only very interested people would bother to do it (physician, Jade HT).

#### Anchoring

Jade HT has recently established a project named Jade Home that is intended to help entrepreneurs with their DRC initiatives. Nevertheless, in large hospitals, it can be difficult to make everyone aware of new services:

I did not know that there was an innovation department until May–June, [. . .] but they have rather helped me a lot afterward to try to spread it out in the clinic [. . .]. I think they have a very important role to support deeply engaged people because along the way, you get very fed up (physician, Jade HT).

From this, we can conjecture that a supportive organisational arrangement will support the idealistic entrepreneur to chase the idea.

#### Anchoring and remote infrastructure

In one of the clinics, an idealistic entrepreneur, a nurse, is involved in 4 different DRC projects. However, in 3 of the projects, the clinic intend to use a platform that is not approved in Jade HT. Additionally, some of the funding has time limitations:

The platform we plan to use is not approved by Jade [. . .]; we depend on finding a solution soon because we must start spending the money by April. [. . .]. And if we do not find it and things are not approved, then we must send the money back, and the project must be terminated. [. . .] but we are now trying to find other solutions (nurse and project manager, Jade HT).

Moreover, the infrastructure in Jade HT is hampering the progress:

Jade HT is probably very special. I know that both Opal HT and Emerald HT have achieved a lot, so I think that Jade HT likes to feel very special and unique and that we have a different infrastructure than many other HTs. I do not know the technical [side], but it certainly makes innovation projects difficult to start (nurse and project manager, Jade HT).

The Jade HT case shows that idealistic entrepreneurship is in place, likewise with organisational anchoring, but there is a problem in integrating the new with the current digital infrastructure.

### Case 3: Emerald HT

Emerald HT is a medium-sized HT and has the largest number of DRC initiatives in production. They have bought a platform they use as a basis for DRC innovation, and have established a DRC programme.

#### Idealistic entrepreneurship and anchoring

In Emerald HT, a physician was a pioneer in DRC through a project that she called ‘the child of my heart’. In collaboration with a platform vendor, she developed a system to follow up on patients with epilepsy. The project manager came from the Hospital Partner. While this project was ongoing, Emerald established a DRC programme (in mid-2020) to align with its technology strategy, where mobility and distance were among the areas of focus. The project manager became the programme manager.

The physician pointed to organisational anchoring and support from committed actors as success factors:

Good anchoring with the management and support from there are completely alpha and omega, that it is highlighted as a priority and that [the management] continuously informs the health personnel group [. . .]. Furthermore, there is a need for some resource persons and drivers who believe in this way of working and who can take the lead. Here, I will point to the programme manager; he was an important resource person in that [early] phase (physician, Emerald HT).

We conjecture that both the programme manager and the physician are idealistic entrepreneurs.

The management has given legitimacy to the DRC programme to encourage further innovation:

[. . .] there are quite long distances between the hospitals and where the patients are. Therefore, we have organised this as a programme and worked systematically in all hospitals, down to the wards and in the clinics (research and innovation director, Emerald HT).

#### Anchoring and remote infrastructure

There are several sources of funding for DRC projects, including public sector organisations (regional, national, and the European Union) and some ideal/private foundations. However, the solution’s survival when the project period is over is uncertain, and there is a struggle for prioritisation in receiving the Hospital Partner’s help with integration issues. For this reason, some HTs have purchased their own platforms to develop sustainable solutions. In relation to the epilepsy project, Emerald HT bought a platform and thus paved the way for new patient groups.

The programme manager in Emerald HT explained that the platform had the functionality that met the needs of many clinics, and its systematic approach was a success factor for spreading its use across clinics.

To use the platform on a new patient group is very easy in terms of process, and we have spent time on fine-tuning the process. We have a clear progression plan and activity plan on everything to be done from A to Z [. . .]. So we standardise the process so that it takes as little time and effort as possible [. . .], so we try to create a good practice for implementation (programme manager, Emerald HT).

In the beginning of 2022, Emerald HT recruited local DRC managers to serve as supervisors. We can interpret this as top management support since Emerald HT has the resources; additionally, the programme manager pinpointed top managers as champions for DRC:

The management challenges the clinics to come up with new proposals for new areas where both video and the type of self-registration can be used. So there is a strong focus on looking for new areas. Then we [in the DRC programme] have been very outgoing and sold this in. [. . .]. And new initiatives come from the clinics, from the professional community itself, when we have shown how this can work, and we refer to others’ experiences, so it is the professional community that is the driver.

This case shows that an idealistic entrepreneur is important in the early phases, but that anchoring and remote infrastructure is needed for successful integration and scaling.

### Summary of the findings

Our findings suggest that the 3 key mechanisms – idealistic entrepreneurship, anchoring and remote infrastructure – must be present to provide an outcome characterised by continuous production of innovative DRC solutions. We summarise our findings in Table 4. We see that in Topaz HT, only idealistic entrepreneurship is present; thus, there is no innovation. Jade HT has both idealistic entrepreneurship and anchoring but lacks remote infrastructure. The lack of strategy hinders initiatives from being started, and if these have been launched, it is problematic to advance from the pilot phase to production; thus, the innovation pace is slow. In Emerald HT, all 3 key mechanisms are in place, and it has a steady stream of innovation projects.

## Discussion

**Figure 2.**
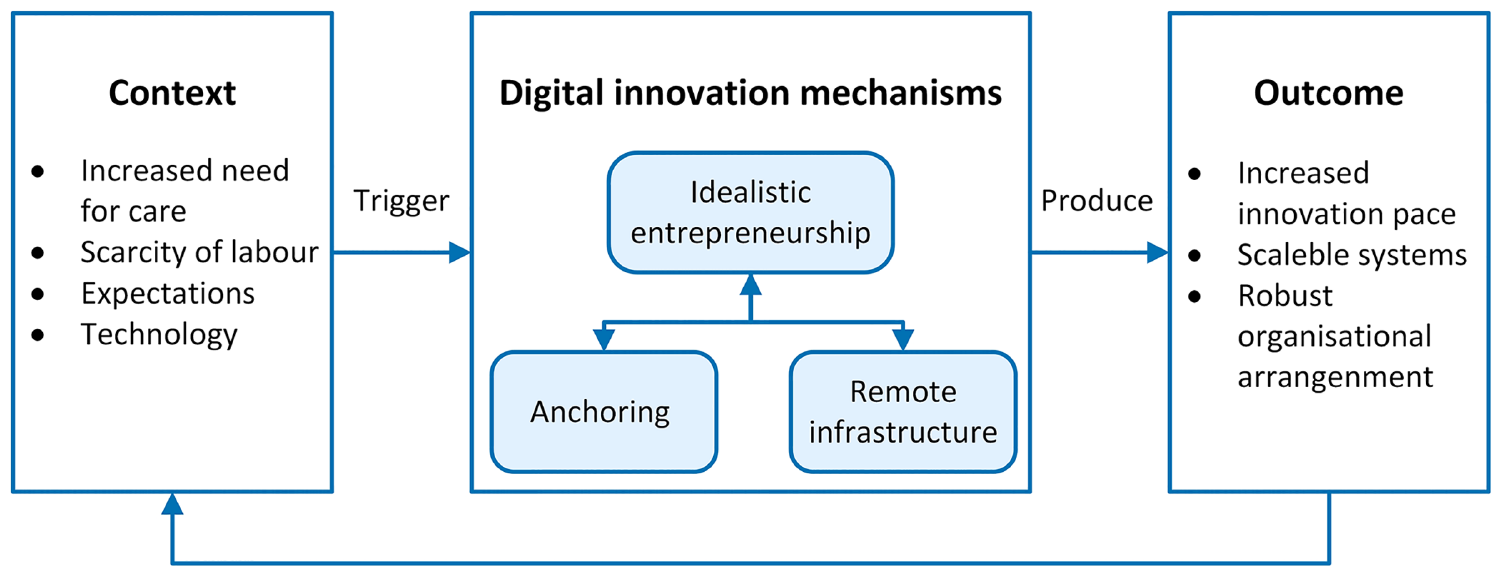
Model showing the interplay among digital innovation mechanisms and their outcomes.

In response to our research question (*How can a DRC initiative be integrated into a large-scale digital infrastructure?*) and based on our findings, we propose a variance model (see Figure 2) inspired by Pawson and Tilley’s^45^ context-mechanism-outcome approach. The model demonstrates the dynamics among the key mechanisms in transforming contextual pressure and expectations into casual concrete outcomes^45^ in large-scale digital infrastructures.^20^ We proceed by discussing our model in relation to prior literature, as well as our new contributions.

*Context* is what triggers the mechanisms and guides the development of how change is formed and facilitated.^45^ The contextual conditions that we assess as the most prominent stem from different layers (individual capacities of actors and stakeholders, interpersonal relationship, institutional setting, and the broader infrastructural and welfare system).^47^ The welfare system in Norway has goals in line with the UN’s sustainability goals, that is, increased life expectancy and a higher number of years of good health for the entire population.^4^ The most recent national plan for hospitals states that more people need healthcare, there is an increasing scarcity of labour, and the Norwegian citizens expect a digital public sector.^4^ The government has put pressure on hospitals to address these issues and take advantage of digital technology to be more efficient and to provide patients with hospital services in their homes and make them active assistants.^4^ At the same time, it is important that the citizens receive equal services across the country, something that emphasises collaboration and coordination.^48^

When some hospitals are successful in their initiatives, such as the epilepsy programme in Emerald HT, this institutional setting can put pressure on other hospitals to facilitate their DIN practices. Likewise, experiences with positive outcomes in a hospital can exert cultural pressure to improve its DIN capability. Moreover, when the organisation establishes dedicated teams, as part of the structure in the line organisation, to advocate and follow up on DRC and innovation, the interpersonal relationship makes innovation practices legitimate and spurs further innovation. The latter is also in line with Henfridsson and Bygstad’s^49^ adoption mechanism, which is activated when new services are developed because more resources are available.

*Idealistic entrepreneurship* is the first key mechanism. It means that many of the DINs come from discoveries or ideas on how to take advantage of the new technology.^30^ These discoveries or ideas are attributed to enthusiastic clinical entrepreneurs or, as Chesbrough (p. 39)^50^ calls them, ‘innovation missionaries’ who work for a cause rather than for financial profit. These entrepreneurs have a substantial work burden and face unexpected obstacles, which may lead to the termination of the initiative.

Additionally, the clinical entrepreneurs have a difficult time understanding why their organisations cannot have several platforms for DRC, and they are dissatisfied with waiting for decisions that sometimes take so long that the funding has expired. However, in such cases, the idealistic entrepreneurs sometimes explore new options to continue the innovative process.^30^

A lesson learned is that the idealistic entrepreneur is unaware of either the organisational or the ICT complexity. Nonetheless, the initiatives have a good starting point when they come from clinicians. Organisational support can mitigate entrepreneur burnout. One measure to support idealistic entrepreneurs is to have an organisational structure that can help with the tasks that are way beyond the scope of the clinical profession’s daily work; clinicians have patient focus and quality of healthcare as their driving forces, not administrative tasks.^51,52^ This organisational structure is part of the second mechanism – anchoring. Importantly, it is often the case that institutional entrepreneurs start the anchoring process by mobilising allies to gain support for the intended change.^34,53^

The *anchoring* mechanism in our model is actualised when DIN related to DRC is a collective effort for the decentralised organisation and when the use of lightweight IT is accepted. The mechanism can be observed in the form of a new organisational structure and investment in DRC solutions from a long-term perspective, instead of single and temporary initiatives.

The establishment of new organisational structures requires top management support. Since DRC is perceived as a remedy to reduce cost, this is aligned with the logic of cost control by healthcare managers,^54,55^ and together with the government’s pressure to transfer treatment to patients’ homes, it can sway the top management to support such initiatives. The central task of the new organisational structure involves supporting and streamlining the process from idea to production. Especially, it is important to support the administrative tasks (eg, legal, risk and vulnerability analysis, General Data Protection Regulation, applying for funding). In comparison, England has created a Health Systems Support Framework (HSSF) to provide the NHS and its partners ‘accredited third-party suppliers with expertise in essential support services ranging from digital infrastructure and advanced analytics, to system transformation and tools to support self-management and the personalisation of care’.^56^ The HSSF includes support related to the administrative tasks mentioned above and for services sorted under DRC.

Furthermore, the new DRC organisation is useful in advocating the possibilities for DRC across clinics. Experiences from other countries show that organisational-wide acceptance of telemedicine must be present to succeed,^57^ and strategies for creating a positive attitude to telemedicine are crucial.^38^ When clinicians recognise its usefulness to their colleagues, they become more interested,^38^ and as ordinary employees, can contribute to innovations. This can be understood as a reinforcing process, in line with Henfridsson and Bygstad’s^49^ innovation mechanism, which is triggered when malleable technological artefacts are recombined to provide new services. Additionally, this interest can be explained by the institutional logics for healthcare personnel in 4 ways. First, it can be because of the care logic, where the patient as a person is at the forefront (will be spared time-consuming and complicated travel).^58^ Second, it can make sense due to a potential increase in performance for the clinicians by serving more patients.^52^ Third, it can apply to the status logic, so the healthcare personnel would avoid being ‘deemed “old-fashioned” if they did not follow the current trend’ (p. 27).^59^ Fourth, it can apply to the logic of personal advantage by recognising it as an opportunity for career advancement.^59^ Nonetheless, we conjecture that the professional community’s interests and actions have brought legitimacy and anchoring to the DRC practice.^60^

Nevertheless, the use of lightweight IT is challenging due to the funding structure for the hospitals and the dependence on the shared ICT operational organisation. The first issue constrains the managers’ ability to make large investments, and the second issue has led to lengthy processes and rejections related to integration. Without financial resources to invest in DRC solutions and the willingness to realise the necessary integration, there can be resistance to using the system because of inefficiency (double registration and checks in other systems).^51^ Suboptimal systems do not fit healthcare managers’ logics of work optimisation and quality of care,^52^ hence, it is not that strange that the initiatives can be blocked with such arguments. Thus, it is essential that the initiative is grounded in analyses that show that DRC is a fruitful way for improvements and is embraced by leaders at management and clinical level.^38,57^ In the end, is a willingness among clinicians to use DRC as an approach for health care services a key factor to success in the long term.^8,39^

Time-limited funding in such a complex environment is also problematic. The projects are sometimes not started due to organisational obstacles, or the initiatives are set aside as the budget will be unavailable.^57,61^ Nonetheless, Topaz HT as the smallest hospital, has less access to human and financial resources compared to the others, which may influence how ICT investments are performed.^62^

The third key mechanism, *remote infrastructure*, enables integration between the DRC solution and the core systems in both hospitals and primary care organisations. Consequently, the remote infrastructure has 3 distinctive characteristics. First, it must fulfil the security requirements of the current digital infrastructure.^63^ Second, it has a flexible architecture where new modules can easily be built^64^ or incorporate services from other partners in the ecosystem, such as Henfridsson and Bygstad’s^49^ scaling mechanism, which is prompted by entering new markets through cooperation with ecosystem partners. Third, it is installed base-friendly (adaptable and cooperative) in relation to the current digital infrastructure.^6,65^ This means that innovators should carefully balance the local and global requirements and respect the technological and socio-cultural legacies.^66^ In sum, it will be aligned with the national vision of increased coordination and collaboration across the health sector. Therefore, we suggest that a remote infrastructure in healthcare should be connected to the digital infrastructure of both hospitals and primary care organisations (eg, the primary care physicians’ electronic health records). Moreover, we consider this important, so patients can avoid the risk of having 2 apps to operate or that healthcare professionals have to do double registration of some kind.

The anchoring and remote infrastructure mechanisms as means to speed up the innovation pace in digital infrastructures are in line with the finding on how to support 2-speed DIN, where lightweight DRC solutions are developed at a higher speed and with a more agile approach compared with the heavyweight core systems.^27^

*The outcome* of well-established DIN mechanisms is an environment with a continuous creation of productive innovations. This implies 3 major achievements. First, the pace of innovation is faster due to the support and empowerment of idealistic entrepreneurship that proper anchoring provides,^34^ in combination with the capabilities of a remote infrastructure. Second, the innovations are scalable in both adding new functionality and being spread across the organisation when they are legitimate or compatible with the sociotechnical particularities of the installed base. This means compatibility with either the organisational practices, (such as using the asthma tuner, which does not require connection to the digital infrastructure to be useful for the patient and the clinician) or the digital infrastructure (such as the Checkware platform ie, able to send information to the electronic health records). Nevertheless, it is a prerequisite that powerful actors embrace new arrangements to change their practices.^53^ Accordingly, if the remote infrastructure is installed base-friendly it may facilitate geographic scaling across the hospital region.^65^ Third, when the DIN mechanisms are in place, organisational learning can shape a robust and legitimate organisational arrangement to embrace new ideas and to make changes.^67^ However, since there always will be newcomers in the organisation, the education aspect must be a continuous part of the DRC initiative.^38^

A limitation of this study is that the model applies to DIN in healthcare settings, but we believe that the model is applicable to large professional user organisations in general. Exploration of the generalisability of the model in other settings and for scaling DRC in general in other countries is an avenue for further research. We are also aware that it is possible to identify other key mechanisms that have equal or even greater impacts on the outcomes. An additional limitation is that the context in our model that influences the mechanisms is based on the current situation; hence, if the context changes, both the mechanisms and the outcomes can vary as well.^68^

## Conclusion

With this case study in the hospital sector, we have illuminated and outlined a variance model to answer our research question of how a DRC initiative can be integrated into a large-scale digital infrastructure. Primary and secondary data are used to explore 72 DRC initiatives at 9 HTs in a large hospital region in Norway. The data showed a pattern regarding an HT’s ability to innovate and scale in a successful manner (see Table 4). We identify 3 key mechanisms that must coexist in a dynamic interplay: idealistic entrepreneurship, anchoring and remote infrastructure. Moreover, contextual pressure from the national strategies and from organisational learning will form and even strengthen the key mechanisms when the outcome is perceived as successful within the organisation. Our contribution to the DIN research stream is a model of DIN mechanisms, leading to a faster-paced creation of productive scalable innovations (see Figure 2). We also contribute to the digital infrastructure stream by showing that to increase innovation through DIN mechanisms in an existing digital infrastructure, the most important actors (managers, clinicians, and IT experts) must both legitimise the relevant IT artefacts and accept the possibility of changes in clinical practices.

For practice, we offer 5 lessons learned to speed up the pace of innovation: (1) Create a DRC organisational structure to support the idealistic entrepreneur. (2) Ensure financial predictability, and (3) secure anchoring upward in the IT governance structure. (4) Make the remote/new infrastructure appropriate for integration with the current digital infrastructure. (5) When success is achieved, advocate the DRC initiative across the organisation to spur others to innovate as well.
